# Influence of PSMB6 and PSMB9 Genetic Polymorphisms on Bortezomib-Based Therapy Response in Newly Diagnosed Multiple Myeloma

**DOI:** 10.7759/cureus.81081

**Published:** 2025-03-24

**Authors:** Nidhi Sood, Abdoul Hamide, Biswajit Dubashi, Mathaiyan Jayanthi, A.K. Munirajan

**Affiliations:** 1 Medicine, Jawaharlal Institute of Postgraduate Medical Education and Research, Puducherry, IND; 2 Medical Oncology, Jawaharlal Institute of Postgraduate Medical Education and Research, Puducherry, IND; 3 Pharmacology, Jawaharlal Institute of Postgraduate Medical Education and Research, Puducherry, IND; 4 Genetics, University of Madras, Chennai, IND

**Keywords:** bortezomib, multiple myeloma, psmb6, psmb9, response rate, survival rate

## Abstract

Background: Bortezomib (BTZ)-based regimens play a crucial role in the treatment of multiple myeloma (MM), significantly improving patient outcomes. BTZ, a proteasome inhibitor, interferes with cellular processes essential for cancer cell growth and survival, resulting in high response rates. Its use in initial and recurrent treatment strategies has been associated with extended disease control and improved long-term outcomes, contributing to overall survival. This study aimed to delineate the clinical characteristics of newly diagnosed MM patients and investigate the influence of single nucleotide polymorphisms (SNPs) in *PSMB6* and *PSMB9 *genes on the risk and response to BTZ-based chemotherapy, in comparison with the control group.

Methods: This prospective study was conducted at Jawaharlal Institute of Postgraduate Medical Education and Research (JIPMER), Pondicherry, India, comprising 92 newly diagnosed MM cases and 92 controls. Patient selection was based on receiving first-line BTZ therapy with available outcome data. DNA was extracted from peripheral blood samples, followed by SNP analysis using real-time polymerase chain reaction (PCR) with appropriate validation and quality control measures. The patients were stratified into good responders and poor responders. The association of genetic polymorphism with the response to BTZ treatment was carried out using chi-square. Survival analysis was conducted using Kaplan-Meier to estimate the overall survival (OS) and progression-free survival (PFS).

Results: In our study, the median age of participants was 55 years, with a predominant 71.7% being males. Backache (66.3%) emerged as the most commonly reported symptom in patients followed by myalgia (53.3%) and bony swelling (12%). The GA+AA genotype in *PSMB6* (rs3169950) was significantly more prevalent in poor responders compared to good responders (odds ratio (OR)=3.20, p=0.011), indicating a potential genetic marker for treatment response. The presence of the GA+AA genotype in *PSMB6* gene rs3169950 was significantly higher among poor responders compared to good responders (OR=3.200, p=0.011). Likewise, the GA+AA genotype of the *PSMB9* gene (rs17587) was predominantly observed among poor responders (OR=3.08, p=0.011). However, no differences were noted in survival analysis.

Conclusion: The findings of this study represent a potential breakthrough, indicating that common genetic variants may significantly influence the development of MM and impact treatment response, providing valuable insights for predicting patient outcomes with BTZ-based therapies.

## Introduction

Multiple myeloma (MM), a multifaceted and refractory hematologic malignancy comprising nearly 10% of all hematologic neoplasms, presents significant therapeutic challenges due to its clonal heterogeneity, chemoresistance, and interpatient variability in treatment response. MM is characterized by the aberrant multiplication of plasma cells within the bone marrow. This unchecked expansion disrupts normal hematopoiesis and leads to systemic organ dysfunction, manifesting as CRAB (i.e., hypercalcemia, renal impairment, anemia, and osteolytic bone lesions) [1,2]. Bone pain affects approximately 70% of MM patients, while infections occur in 75%, increasing susceptibility to complications. Renal failure impacts 25% of patients, and neurological symptoms are observed in certain cases. Anemia is prevalent in 80% of patients [3]. According to the Global Cancer Observatory (GLOBOCAN) and data from the International Agency for Research on Cancer (IARC), the global occurrence of newly diagnosed MM (NDMM) is 160,000 with a mortality of 106,000 in 2018. In comparison to Western countries, MM incidence is lower in India, though genetic factors influencing treatment response remain understudied in this population [4,5].

A crucial cellular mechanism for degrading and recycling proteins that are vital for maintaining cellular homeostasis and regulating numerous cellular processes is called the ubiquitin-proteasome pathway (UPP) [6]. This pathway is leveraged by the bortezomib (BTZ), a proteasome inhibitor (PI), in treating MM by selectively binding to the 26S proteasome, hindering its function, and averting the degradation of ubiquitin-tagged proteins. This causes the accumulation of misfolded or damaged proteins, resulting in apoptosis in malignant plasma cells and contributing to BTZ cytotoxic effects against MM cells [7-9].

Genetic variations in proteasome subunit genes, specifically proteasome 20S subunit beta 6 (*PSMB6*) and proteasome 20S subunit beta 9 (*PSMB9*), are essential as they impact proteasome activity and cellular responses to BTZ. These variations can alter protein degradation pathways, impacting myeloma cell sensitivity to BTZ and thereby influencing disease progression and treatment efficacy [10,11]. Owing to the genes’ significant role in proteasome function and association with MM risk, these genes are chosen. This study emphasizes the role of *PSMB6 *and *PSMB9* in MM patients treated with BTZ-based chemotherapy, as these genes encode proteasome subunits involved in drug resistance. Understanding their molecular mechanisms, such as alterations in proteasome function or stress response pathways, could lead to personalized treatment strategies, improving efficacy and patient outcomes [12-15].

Given the conflicting reports on the role of genetic variants in MM pathogenesis, this study aims to clarify the association between rs2304975 and rs3169950 in the *PSMB6* gene, and rs17587 in the *PSMB9* gene, with MM susceptibility and progression. It also explores the association of single nucleotide polymorphisms (SNPs) with treatment response to BTZ and survival in MM patients.

This article was previously presented in an international conference on “Trends in Biological Sciences: Impetus on Human Health (ICBS)” held on 12-13th October 2023 at SRM Arts and Science College, Kattankulathur, Tamil Nadu.

## Materials and methods

Study design and setting

This prospective, case-control genetic study, designed to assess the impact of genetic polymorphisms on MM response to BTZ, was conducted from January 2018 to December 2021. It was carried out by the Department of Medicine in collaboration with the Department of Medical Oncology at the Jawaharlal Institute of Postgraduate Medical Education and Research (JIPMER), India.

Ethical consideration

The study received approval from the Jawaharlal Institute of Postgraduate Medical Education and Research, Puducherry, Institutional Ethics Committee (JIP/IEC/2017/0152), ensuring that all research protocols adhered to ethical standards for patient safety and confidentiality. The study adhered to the principles outlined in the Declaration of Helsinki and followed Good Clinical Practice (GCP) guidelines. Participants were enrolled based on specific eligibility criteria, and informed consent was obtained from all individuals to be a part of the study.

Inclusion and exclusion criteria

Under the inclusion criteria of NDMM patients on first-line BTZ therapy with available outcome data, 92 patients were enrolled. Patients with monoclonal gammopathy of undetermined significance (MGUS) or solitary plasmacytoma were excluded. A total of 92 healthy controls were included, with the exclusion of individuals having prior cancer to eliminate confounding factors.

Sample size

The sample size was calculated using PS Power and Sample Size Calculations software (version 3.0; Vanderbilt University, Nashville, Tennessee, USA). A total of 92 cases and 92 controls were included, providing 80% power to detect an odds ratio (OR) of 3.5 with a type I error probability (α) set at 0.05. The total sample size for determining allele and genotype frequencies of BTZ-related gene polymorphisms was 184. According to the recruitment criteria, the number of groups were divided. Group I includes patients of all age groups who were diagnosed with MM and have a good response toward BTZ treatment; Group II includes patients of all age groups who were diagnosed with MM and have a poor response toward BTZ treatment; Group III includes healthy individuals above 30 years of age.

Sample collection

All patients have undergone minimum induction chemotherapy of four to six cycles, and their responses were assessed. Peripheral venous blood (five milliliters) was collected from each patient and centrifuged at 2500 rpm for five minutes at 4°C. The plasma was separated and discarded from the whole blood and the buffy coat was stored at -80°C until DNA extraction.

SNP

These SNPs (*PSMB6* C>T [rs2304975], *PSMB6* G>A [rs3169950], and *PSMB9* G>A [rs17587]) were selected for their role in proteasome activity, potential BTZ resistance, and association with MM progression. Their allele and genotype frequencies adhered to Hardy-Weinberg equilibrium (HWE) as shown in Appendices B and C, confirming the absence of selection bias and genotyping errors, thereby validating the study's findings [16].

Sample processing

Genomic DNA was extracted from WBCs using the manual phenol-chloroform method. The extracted DNA was stored at -20°C until genotyping. The quality and quantity of DNA were assessed using a NanoDrop 2000 spectrophotometer (Thermo Fisher Scientific, Waltham, MA, USA) following the manufacturer’s instructions. DNA purity was evaluated with acceptable ranges of 1.8-2.0 for A260/A280, indicating high-purity DNA. DNA samples were diluted to a final concentration of 50 ng/μL, incubated at 37°C for 12 hours, and subsequently stored at 4°C overnight prior to genotyping.

Genotyping was performed using TaqMan SNP assay kits (Applied Biosystems, Foster City, CA, USA) following the manufacturer’s protocol. The TaqMan-probe assay was used to genotype three SNPs: *PSMB6* C>T [rs2304975], *PSMB6* G>A [rs3169950], and *PSMB9* G>A [rs17587]. Real-time polymerase chain reactions (RT-PCR) were conducted in duplicate on a 96-well plate using an AB 7300 system (Applied Biosystems). The PCR reaction was carried out in a final volume of 10 µL, consisting of 2.5 µL of extracted DNA, 5 µL of TaqMan master mix, 0.25 µL of TaqMan assay reagent, and 2.25 µL of Milli-Q water. Table 1 provides comprehensive details on the selected SNPs.

**Table 1:** SNPs and corresponding genotyping assay kits SNP: Single nucleotide polymorphism; Missense variant: A point mutation in which a single nucleotide change results in the substitution of one amino acid for another in the protein sequence; Synonymous variant: Silent mutation that occurs in the coding region of a gene but does not change the resulting amino acid sequence of the protein

S. No.	Gene Name	rs ID	Nucleotide Substitution	Position in Chromosome	SNP Location	Amino Acid Substitution	Assay ID
1	PSMB6	rs2304975	C>T	Chr 17:4797724	Missense variant	p.Ser115Arg	C_15976146_30
2	PSMB6	rs3169950	G>A	Chr 17:4796257	Synonymous variant	p.Ala21	C_1217396_10
3	PSMB9	rs17587	G>A	Chr 6:32857313	Missense variant	p.Arg60His	C_8849004_1

The RT-PCR protocol was standardized, beginning with an initial incubation at 50°C, followed by enzyme activation at 95°C for 10 minutes. DNA amplification was carried out through 40 cycles of denaturation at 92°C for 15 seconds, followed by annealing and extension at 60°C for 60 seconds, ensuring optimal reaction efficiency and reproducibility. The International Myeloma Working Group (IMWG) criteria were followed for response evaluation [17]; then, the response was classified as good responder (stringent complete response (sCR), complete response (CR), and very good partial response (VGPR)) and poor responders (partial response (PR), stable disease (SD), and progressive disease (PD)).

Data obtained from the patient’s medical records

The patient's medical records were examined to gather demographic, clinical, and laboratory information, including age, gender, comorbidities such as diabetes mellitus, hypertension, renal failure, bone lesions, and Eastern Cooperative Oncology Group performance status (ECOG-PS). Additional laboratory data collected included routine biochemical tests, serum-free light chain (sFLC) levels, serum protein electrophoresis/immunofixation results, staging information, therapeutic details (induction regimen), and outcomes (post-induction response and survival data). A comprehensive biological evaluation was conducted in this study, encompassing platelet counts, serum calcium, complete blood count (CBC), serum creatinine, serum electrolytes, lactate dehydrogenase (LDH), beta-2 microglobulin, and serum albumin.

The duration from the initiation of treatment to the occurrence of disease progression or death from any cause was defined as progression-free survival (PFS). Overall survival (OS) was defined as the period from the start of treatment until death from any cause.

Statistical analysis

The data were analyzed using IBM SPSS Statistics for Windows, Version 19.0 (Released 2017; IBM Corp., Armonk, New York, United States). Through direct counting, genotyping and allele frequencies in cases as well as controls were investigated. Further, they were tested for HWE by employing the chi-square test, which also computes confidence intervals (CIs) of 95% and OR. Quantitative data were presented as the mean ± standard deviation. Kaplan-Meier survival analysis was employed to assess the association of SNPs with treatment response and survival outcomes. A p-value less than 0.05 was considered to indicate statistical significance.

## Results

The patient cohort consisted of 66 males (72%) and 26 females (28%), with ages ranging from 33 to 75 years and a mean age of 55.24 ± 9.39 years. A healthy control group of 92 individuals was included with an average age of 49.98 ± 8.9 years. The control group comprised 62 (67.4%) males and 30 (32.6%) females. The median duration of symptoms among the patients was three months, ranging from 1 to 24 months. The baseline details of patients and controls are provided in Appendix A.

Co-morbid conditions observed among patients included hypertension in 12 individuals (28%) and diabetes in nine individuals (21%). Additionally, nine patients (10%) were overweight or obese. The prevalent symptoms were backache in 61 patients (66.3%), myalgia in 49 patients (53.3%), and fever in 18 patients (19.6%), respectively. Other symptoms were cough with expectoration, difficulty in walking, bony swelling, headache, and diplopia, observed at lower frequencies, as detailed in Table 2. Plasmacytoma and paraparesis were detected in 20 patients (21.7%) and 18 patients (19.6%). IgGκ is the common myeloma subtype 39 (44.8%), with 10 (11.5%) of patients having light chain disease (LCD). A median of four chemotherapy cycles (range 1-6) was received by the patients. The overall response rate (RR) was 50 (54.3%) and 42 (45.7%) for good responders and poor responders, respectively. Patients were divided into three groups based on the international staging system: stage I: 25 patients (28%), stage II: 23 patients (26%), and stage III: 42 patients (46%). The median neutrophil-lymphocyte ratio (NLR) was found to be 2 (0.5-13). Patients exhibited CRAB features, i.e., hypercalcemia in 19 (22%), renal failure in eight (8.7%), anemia in 63 (68.5%), and bone lesions in 42 (46%) at the time of diagnosis as explained in Table 3. The mean values for beta-2 microglobulin and serum albumin were 5.68 ± 4.75 mg/L and 3.33 ± 0.74 g/dL, respectively.

**Table 2:** Baseline demographic characteristics of patients with MM IgGĸ: Immunoglobulin G kappa; IgGλ: Immunoglobulin G lambda; IgAĸ: Immunoglobulin A kappa; IgAλ: Immunoglobulin A lambda; LCD: Light chain disease; IgG/IgA: Immunoglobulin G and Immunoglobulin A; TB: Tuberculosis; BMI: Body mass index; COPD: Chronic obstructive pulmonary disease; MM: Multiple myeloma; ECOG: Eastern Cooperative Oncology Group

S. No.	Variable	Category	Cases n (%)
1	Gender (n=92)	Male	66 (72)
		Female	26 (28)
2	Type of symptoms (n=92)	Backache	61 (66.3)
		Myalgia	49 (53.3)
		Fever	18 (19.6)
		Cough and expectoration	17 (18.5)
		Difficulty in walking	11 (12)
		Bony swelling	11 (12)
		Headache	2 (2.2)
		Diplopia	1 (1.1)
3	Performance status (n=77) (ECOG)	0	1 (1.3)
		1	42 (54.5)
		2	26 (33.8)
		3	6 (7.8)
		4	2 (2.6)
4	Type of myeloma (by immunofixation) (n=87)	Non-secretory	2 (2.2)
		Secretory	90 (97.8)
		IgGĸ	39 (44.8)
		IgGλ	18 (20.7)
		IgAĸ	11 (12.6)
		IgAλ	6 (7)
		LCD	10 (11.5)
		IgG/IgA	3 (3.4)
5	Para paresis (n=92)	Yes	18 (19.6)
6	Plasmacytoma (n=20)	Bony	18 (90%)
		Extramedullary	2 (10%)
7	BMI (n=92)	Underweight (<18.4)	11 (12.2)
		Normal weight (18.5-24.9)	56 (62.2)
		Overweight (25.0-29.9)	14 (15.6)
		Obese (>30)	9 (10.0)
8	Co-morbidity (n=43)	Hypertension	12 (28)
		Diabetes mellitus	9 (21)
		Heart failure	3 (7)
		TB	3 (7)
		Renal failure	1 (2.3)
		COPD	1 (2.3)
		More than one	14 (32.4)

**Table 3:** Laboratory features of patients with MM (n=92) MM: Multiple myeloma

S. No.	Variable	Category	Cases n (%)/Mean±SD
1	Hypercalcemia (n=92)	Serum calcium (>10.5 mg/dL)	19 (22)
2	Renal failure (n=92)	Serum creatinine (>2 mg/dL)	8 (8.7)
3	Anemia (n=92)	Hemoglobin (Hb <10 g/dL)	63 (68.5)
4	Skeletal fracture (n=92)	Present	42 (46)
5	Hemoglobin (Hb) (n = 92)	Mean ± SD (g/dL)	9.23±2.13
6	Neutrophil-to-lymphocyte ratio (NLR) (n = 92)	Mean ± SD	2.87±2.18
7	Platelet-to-lymphocyte ratio (PLR) (n = 92)	Mean ± SD	2.30±3.90
8	Serum albumin (n = 89)	Mean ± SD (g/dL)	3.33±0.74
9	Serum lactate dehydrogenase (LDH) (n = 50)	Mean ± SD (mg/dL)	343.86±220.33
10	M-value (n = 70)	M-protein (mg/dL)	4.06±2.43
11	Serum free light chain assay (n = 92)	Mean kappa value (mg/L)	185.24±222.37
		Mean lambda value (mg/L)	88.48±167.32
		Mean kappa/lambda ratio	52.17±101.89
12	Beta-2 microglobulin (n = 84)	mg/L (range: lower-higher)	3.88 (1.04-20.70)
13	International Staging System (ISS) (n = 90)	Stage I	25 (28)
		Stage II	23 (26)
		Stage III	42 (46)

Genotyping assessment

The genotyping frequencies of *PSMB6 *(rs2304975, rs3169950) and *PSMB9 *(rs17587) gene polymorphisms were in HWE. In cases, the G allele has a 114 (61%) frequency for* PSMB6* C>T rs2304975, whereas the A allele has a 70 (39%) frequency. In 36 (39.1%) of individuals, the heterozygous CT genotype was present with 39 (42.4%) homozygous CC and 17 (18.5%) TT genotypes. As depicted in Table 4, the allele and genotype frequencies in controls signified significant differences from those recorded in the Indian Telugu in the UK (ITU) subpopulation when analogized to the 1000 Genome project.

**Table 4:** Allele and genotype frequencies of genes PSMB6 and PSMB9 compared to 1000 genome population SNP: Single nucleotide polymorphism; N: Sample size; AFR: African; AMR: American; EAS: East Asian; EUR: European; SAS: South Asian; BEB: Bengali in Bangladesh; GIH: Gujarati Indian in Houston, TX; ITU: Indian Telugu in the UK; PJL: Punjabi in Lahore, Pakistan; STU: Srilankan Tamil in the UK * indicates statistically significant p-value.

SNP	Our Study	AFR	AMR	EAS	EUR	SAS				
						BEB	GIH	ITU	PJL	STU
N	92	661	347	504	503	86	103	102	96	102
*PSMB6 *rs2304975 C>T										
CC	51.0	98.9	79.5	26.4	86.1	38.4	45.6	37.3	41.7	33.3
CT	36.0	1.1	18.7	51.0	13.	43.0	45.6	40.2	43.8	50.0
TT	13.0	-	1.7	22.6	0.8	18.6	8.8	26.5	14.5	16.7
C	69.0	99.5	88.9	51.9	92.6	59.9	68.4	53.4	63.5	58.3
T	31.0	0.5	11.1	58.1	7.4	40.1	31.6	46.6*	36.5	41.7
*PSMB6* rs3169950 G>A										
GG	50.0	58.2	34.0	81.5	34.4	66.3	47.6	63.7	55.2	60.8
GA	39.0	36.2	50.4	17.3	48.7	25.6	44.6	32.4	32.3	35.3
AA	11.0	5.6	15.6	1.2	16.9	8.1	7.8	3.9	12.5	3.9
G	69.5	76.3	59.2	90.2	58.7	79.1	69.9	79.9	71.4	78.4
A	30.5	23.7	40.8	9.8	41.3	20.9*	30.1	20.1	28.6*	21.6
*PSMB9* rs17587 G>A										
GG	59.8	58.7	60.2	61.5	55.3	61.6	62.1	64.7	66.7	70.6
GA	32.6	36.2	34.9	34.5	38.2	32.6	35.0	31.4	30.2	24.5
AA	7.6	5.1	4.9	4.0	6.5	5.8	2.9	3.9	3.1	4.9
G	76.0	76.8	77.7	78.7	74.4	77.9	79.6	80.4	81.8	82.8
A	24.0	23.2	22.3	21.2	25.6	22.1	20.4	19.6	18.2	17.2

The allele frequencies for *PSMB6* G>A rs3169950 among cases were as follows: (G) 106 (57.6%) and (A) 78 (42.4%). The genotype frequencies for this variant were GG:35 (38%), GA:36 (39%), and AA:21 (23%). In the control group, the allele frequencies differed from those observed in the South Asian subpopulations (Bengali in Bangladesh (BEB) and Punjabi in Lahore (PJL)) (Table 4).

Within the cases, the *PSMB9* G>A rs17587 genotype distribution revealed a predominance of the GG variant at 45.6% (42 cases), closely followed by the GA genotype at 44.6% (41 cases), while the AA genotype appeared less frequently, comprising 9.8% (nine cases). The corresponding allele frequencies were G:125 (68%) and A:59 (32%). In the control group, the allele and genotype frequencies closely aligned with those reported in the general population, as documented in the dbSNP database, National Center for Biotechnology Information (NCBI) [18]. Detailed data are presented in Table 4.

**Figure 1:**
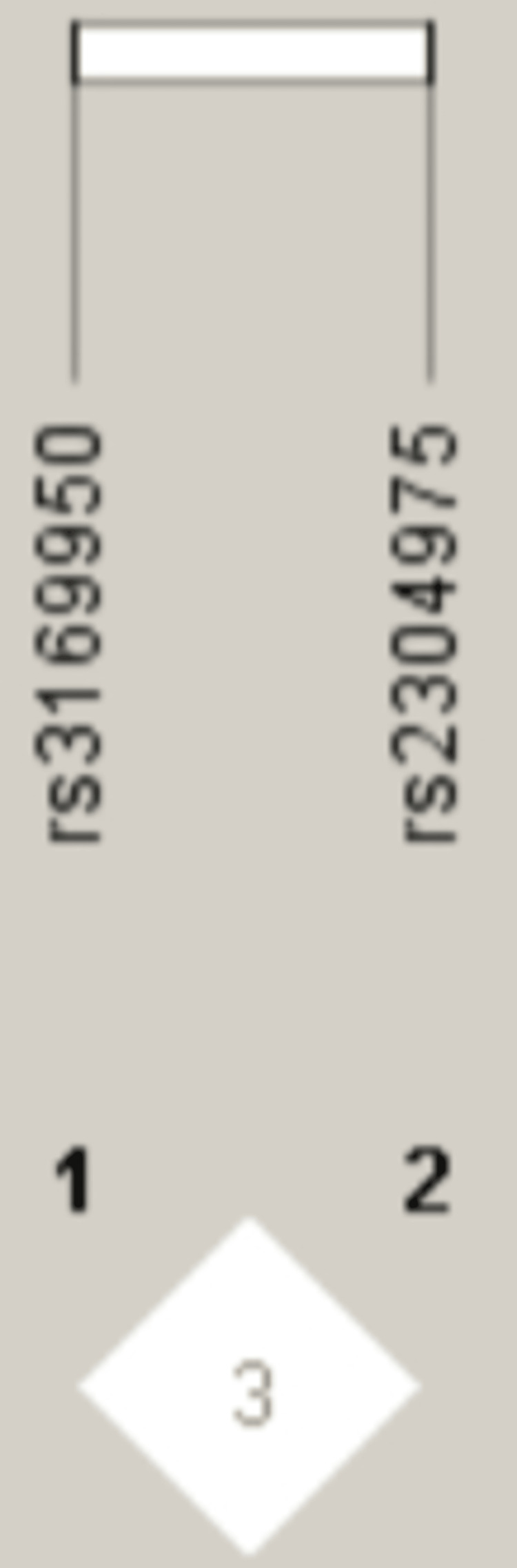
Linkage Disequilibrium (LD) plot of rs3169950 and rs2304975 in the PSMB6 gene among the healthy population PSMB6: Proteasome 20S subunit beta 6

**Figure 2:**
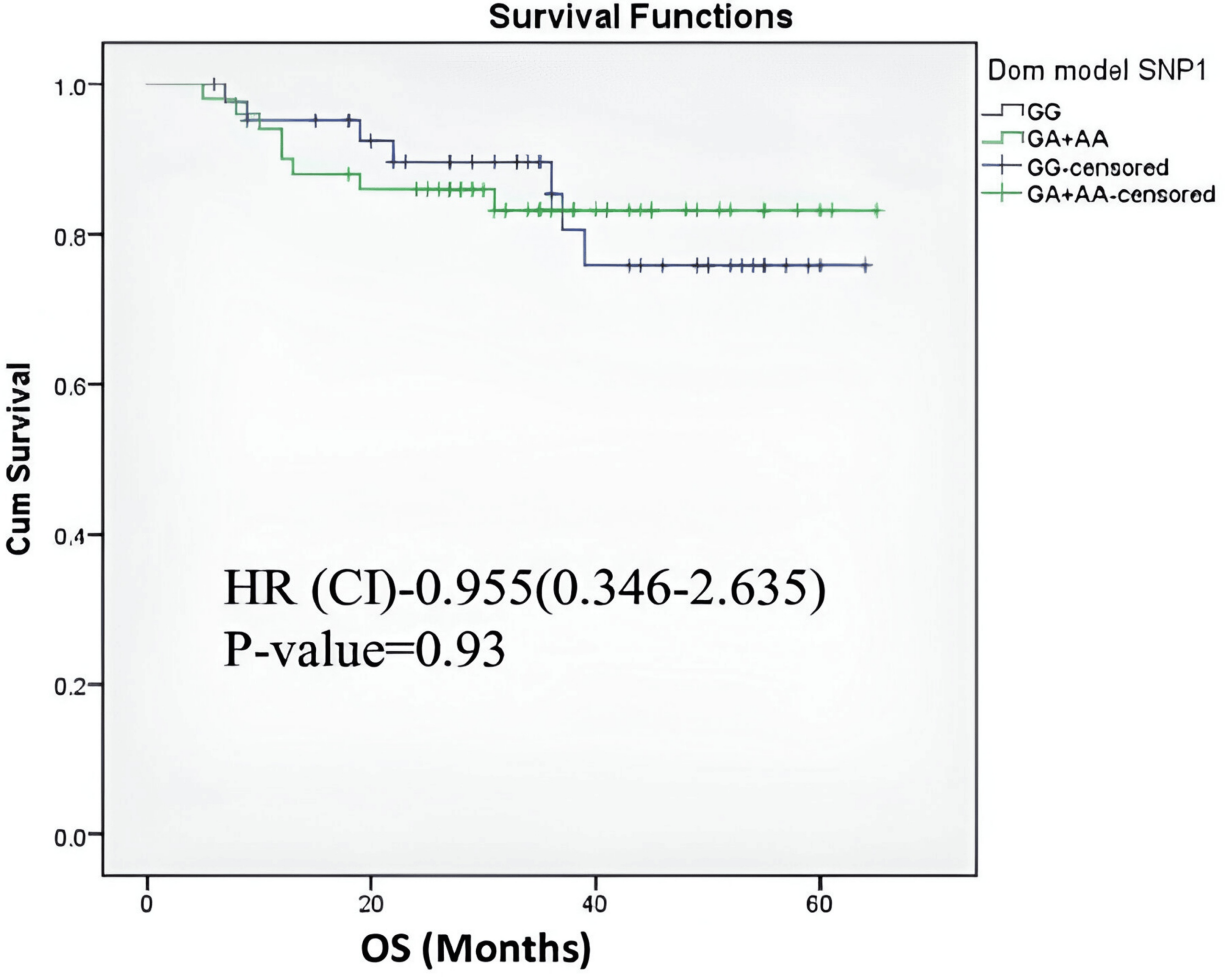
Kaplan-Meier curves showing overall survival (OS) based on the PSMB9 (rs17587) genetic variant, using a genotyping model comparing GG vs. GA+AA HR: Hazard ratio; PSMB9: Proteasome 20S subunit beta 9

In the case-control study, *PSMB9* gene variant rs17587 was significantly associated with MM development (OR=3.053, p-value=0.013). Nevertheless, as detailed in Table 5, the *PSMB6* gene illustrated no such association with MM risk. Figure 1 illustrates that no significant differences between SNPs rs2304975 and rs3169950 (D’=0.03, r^2^=0.0) were indicated by the Linkage Disequilibrium (LD) analysis of the *PSMB6* gene on chromosome 17. Moreover, Figure 2 depicts that no significant associations between *PSMB9* polymorphisms and OS were shown by the Kaplan-Meier survival analysis.

**Table 5:** Case control analysis of the genotypes of the studied polymorphisms PSMB6: Proteasome 20S subunit beta 6; PSMB9: Proteasome 20S subunit beta 9; OR: Odds ratio; CI: Confidence interval

S. No.	Gene	rs ID	Controls (n=92)		Cases (n=92)		p-value	OR (95% CI)
			GG	GA/AA	GG	GA/AA		
1.	PSMB6	rs2304975	47 (51.1)	45 (48.9)	39 (42.4)	53 (57.6)	0.73	1.155 (0.244-1.397)
		rs3169950	46 (50)	46 (50)	35 (38)	57 (62)	0.05	2.327 (0.503-2.654)
2.	PSMB9	rs17587	55 (59.8)	37 (40.2)	42 (45.6)	50 (54.4)	0.013	3.053 (1.262-7.388)

The impact of *PSMB6* and *PSMB9* genetic variants on BTZ-centered therapy response was thoroughly evaluated in this study by utilizing a genetic methodology analysis, namely codominant, dominant, and recessive methodologies. A significant association with MM treatment response was revealed by the SNPs in *PSMB6* rs3169950 and *PSMB9* rs17587 (Table 6). Individuals with the heterozygous mutant genotype in *PSMB6* rs3169950 were more likely to be poor responders (OR=3.200, CI=1.300-7.877, p-value=0.011). Likewise, with the *PSMB9* gene variant rs17587, a comparable pattern was noted. When compared with the GG genotype (OR=3.081, CI=1.301-7.296, p-value=0.011), a poorer response was exhibited by the patients with the mutant GA and AA genotypes.

**Table 6:** Comparison of gene polymorphisms of PSMB6 and PSMB9 with response to bortezomib-based chemotherapy in patients with multiple myeloma 1: Used for reference; OR: Odds ratio; CI: Confidence interval; PSMB6: Proteasome 20S subunit beta 6; PSMB9: Proteasome 20S subunit beta 9

Genotype Model	Good Responder (N=50)	Poor Responders (N=42)	Odds Ratio	95% CI	P-value
1. *PSMB6* rs2304975					
CC	22 (44)	17 (40.5)	1	-	-
CT	19 (38)	17 (40.5)	-	-	-
TT	9 (18)	8 (19)	-	-	-
CC vs CT	19	17	1.158	0.466-2.878	0.752
CC vs TT	9	8	1.150	0.367-3.609	0.810
CC vs CT+TT	28	25	1.155	0.503-2.654	0.733
CC + CT vs TT	41	34	0.933	0.325-2.680	0.897
2. *PSMB6* rs3169950					
GG	25 (50)	10 (23.8)	1	-	-
GA	15 (30)	21 (50)	-	-	-
AA	10 (20)	11 (26.2)	-	-	-
GG vs GA	15	21	3.500	1.303-9.404	0.013
GG vs AA	10	11	2.750	0.891-8.491	0.079
GG vs GA+AA	25	32	3.200	1.300-7.877	0.011
GG + GA vs AA	40	31	0.705	0.265-1.870	0.482
3. *PSMB9* rs17587					
GG	29 (58)	13 (31)	1	-	-
GA	15 (30)	26 (62)	-	-	-
AA	6 (12)	3 (7)	-	-	-
GG vs GA	15	26	3.867	1.553-9.626	0.004
GG vs AA	6	3	1.115	0.241-5.164	0.889
GG vs (GA+AA)	21	29	3.081	1.301-7.296	0.011
(GG + GA) vs AA	44	39	1.773	0.415-7.568	0.439

## Discussion

MM is a hematologic malignancy characterized by the uncontrolled proliferation of plasma cells, often leading to various clinical manifestations, including bone pain. Understanding the prevalence and distribution of bone pain in MM patients is crucial for early diagnosis and effective management. In our study, back pain was the most frequently reported symptom, affecting 66.3% of patients. This finding highlights the importance of skeletal involvement in MM and is consistent with the study by Yong et al., which reported that 61% of patients experienced bone pain [19]. When compared with the findings of Nador et al., which demonstrated that 59% of MM patients reported bone pain at the time of diagnosis [20]. The comparison study by Seesaghur et al. revealed that 47.5% reported baseline bone pain, with the back being primarily affected in 33.7% of cases and other joints in 17.3% [21]. Contrastingly, renal dysfunction was observed in 20% of patients when compared to this study in which the prevalence is 8.7% [19]. Notably, the study by Yong et al. had a much larger sample size (n=4997) and was an observational chart review conducted at various treatment stages. Data were collected from multiple European countries, including Belgium, France, Germany, Italy, Spain, Switzerland, and the UK. However, differences in patient selection, treatment timing, and regional variations in disease presentation and management may account for discrepancies. Since our study focused on newly diagnosed patients and monitored treatment responses, the lower prevalence of renal dysfunction may reflect early intervention and improved supportive care strategies.

In our study, *PSMB6* SNP rs2304975 showed no association, while the rs3169950 GA genotype was significantly linked to poor response to BTZ-based chemotherapy. Additionally, no significant association was observed between these polymorphisms (rs2304975, rs3169950) and survival. Similarly, Lichter et al. found no notable link between *PSMB6* polymorphisms and response to BTZ-based therapy. Their study, which analyzed 76 DNA samples from relapsed MM patients, evaluated treatment responses to BTZ and dexamethasone before and after therapy. However, they reported that the A allele of rs3169950 was linked to shorter OS compared to the G allele [22]. Differences in sample size, patient population, and treatment regimens may explain the variations in findings. Given the limited research on *PSMB6* polymorphisms in MM, further studies are warranted to better understand their role in treatment outcomes.

The higher frequency of GA and AA genotypes in cases (54.4%) compared to controls (40.2%) suggests a potential link to MM susceptibility. However, as *PSMB9* SNP rs17587 showed no significant deviation from the 1000 genomes project, further investigation is needed to determine if this reflects a true risk factor or population genetic variation. Compared to Ozbas-Gerceker et al., who reported lower GA (44%) and AA (4%) frequencies in MM cases (n=25, p=0.028) within the Southeastern Anatolian population, our study shows a higher prevalence. These differences may be due to variations in sample size, ethnicity, and genetic predisposition. Ozbas-Gerceker et al.'s findings suggest a possible role of these polymorphisms in MM risk, highlighting the need for further large-scale studies to better understand their impact across different populations [23].

With a growing number of novel polymorphisms over time, MM cells exhibit hyperactivation of DNA repair mechanisms, granting them a survival advantage and drug resistance. Identifying genetic abnormalities is crucial, as they play a significant role in the pathogenesis and progression of active MM. In particular, *PSMB6* and *PSMB9* polymorphisms may influence proteasome function and response to treatment, making them potential targets for therapeutic intervention. This underscores the importance of targeting these early genetic events to develop more effective, personalized treatment strategies for MM [24,25].

Approved by the US FDA in 2003, BTZ has become a cornerstone in MM treatment by inducing apoptosis through PI. It serves as the backbone of effective three-drug regimens, significantly improving OS, PFS, and RRs [26]. In combination with dexamethasone and other chemotherapeutic agents, BTZ achieves over 50% RRs in NDMM/relapsed patients, with frontline therapy yielding response rates of 80-90% [27,28]. The UPP plays a key role in tumorigenesis, making it a crucial target for MM treatment with BTZ. Its regulation of protein degradation is vital in myeloid malignancies, and ongoing research continues to uncover its impact on cancer progression [29,30].

Genetic variations in proteasome subunits, such as *PSMB6* and *PSMB9* polymorphisms, may significantly influence BTZ sensitivity and resistance. These SNPs can alter the structure, function, or expression of proteasomal subunits, potentially affecting BTZ binding affinity and subsequent PI efficiency [14]. Genetic variations in *PSMB6* rs3169950 and *PSMB9* rs17587 influence proteasome activity, affecting protein degradation, UPP regulation, and MM treatment response. This study highlights their role in BTZ sensitivity and resistance, underscoring the value of genotype-guided treatment. Identifying these polymorphisms could help tailor therapy by predicting response, guiding dose adjustments, and selecting alternative PIs or combination regimens to improve efficacy and minimize toxicity. Integrating genetic profiling into MM management may enhance personalized treatment strategies and optimize patient outcomes.

However, this study had limitations, including its narrow focus on specific genes and polymorphisms, while other genetic variants and pathways may also contribute to treatment outcomes. A genome-wide approach could provide a more comprehensive understanding of the genetic landscape in MM. Additionally, plasma concentrations of the drug and its metabolites were not measured, which could have offered further insights into pharmacokinetics, pharmacodynamics, and overall drug efficacy.

## Conclusions

In conclusion, the study findings suggested that in the *PSMB9* variant rs17587, patients with GA and AA genotypes showed a poorer response compared to the GG genotype (OR=3.081, p=0.011). Identifying *PSMB6* and *PSMB9* SNPs offers insights into BTZ response, but further research with larger, diverse populations is needed to validate findings, understand genetic influences, and improve personalized MM treatment. Personalized therapy in MM could significantly enhance patient outcomes by integrating genetic profiling into treatment planning. By identifying *PSMB6* and *PSMB9* polymorphisms linked to BTZ response, clinicians can tailor drug selection, dosage, and combination strategies to maximize efficacy and minimize toxicity. Patients with genetic variants associated with poor response or resistance may benefit from alternative PIs, dose modifications, or adjunct therapies to improve treatment effectiveness. This approach not only reduces unnecessary side effects but also enhances PFS and overall prognosis, ultimately leading to more precise and effective MM management.

## Appendices

Appendix A 

**Table 7:** Baseline demographic characteristics among MM patients and controls BMI: Body mass index; MM: Multiple myeloma

S. No.	Variable	Frequency (%)	
		Cases (n=92)	Controls (n=92)
1	Gender		
	Male	66 (71.7)	62 (67.4)
	Female	26 (28.3)	30 (32.6)
2	Age		
	30-45 yr	15 (16.3)	24 (26)
	46-59 yr	39 (42.4)	49 (53.3)
	>60 yr	38 (41.3)	19 (20.7)
3	BMI		
	Underweight (<18.4)	11 (12.2)	5 (5.4)
	Normal weight (18.5-24.9)	56 (62.2)	70 (76.1)
	Overweight (25.0-29.9)	14 (15.6)	11 (12.0)
	Obese (>30)	9 (10.0)	6 (6.5)
4	Smoking status	14 (15.2)	12 (17.4)
5	Geographical location/domicile		
	Cuddalore	24 (26.1)	10 (10.9)
	Puducherry	17 (18.5)	68 (73.9)
	Villupuram	15 (16.3)	9 (9.8)
	Others	36 (39.1)	5 (5.5)
6	Distance travelled from home (<100 km)	57 (62)	90 (97.8)
	101-200 km	12 (13)	2 (2.2)
	>201 km	23 (25)	0

Appendix B 

**Table 8:** Genotype and allele distribution of the studied gene polymorphism in controls (N=92) The genotype and allele frequencies are in Hardy-Weinberg equilibrium (HWE). SNP: Single nucleotide polymorphism

rs ID	SNP	Genotype Frequency (%)	Allele Frequency (%)	HWE (p-value)
rs17587	*PSMB9* G>A	GG 55 (59.8)	G 140 (76)	0.31
		GA 30 (32.6)	A 44 (24)	
		AA 7 (7.6)		
rs2304975	*PSMB6* C>T	CC 47 (51)	C 127 (69)	0.12
		CT 33 (36)	T 57 (31)	
		TT 12 (13)		
rs3169950	*PSMB6* G>A	GG 46 (50)	G 128 (69.5)	0.46
		GA 36 (39)	A 56 (30.5)	
		AA 10 (11)		

Appendix C 

**Table 9:** Genotype and allele distribution of the studied gene polymorphism in multiple myeloma patients (N=92) The genotype and allele frequencies are in Hardy Weinberg equilibrium (HWE). SNP: Single nucleotide polymorphism

rs ID	SNP	Genotype Frequency (%)	Allele Frequency (%)	HWE (p-value)
rs17587	*PSMB9* G>A	GG 42 (45.6)	G 125 (68)	0.82
		GA 41 (44.6)	A 59 (32)	
		AA 9(9.8)		
rs2304975	*PSMB6* C>T	CC 39 (42.4)	C 114 (61)	0.1
		CT 36 (39.1)	T 70 (39)	
		TT 17 (18.5)		
rs3169950	*PSMB6* G>A	GG 35 (38)	G 106 (57.6)	0.06
		GA 36 (39)	A 78 (42.4)	
		AA 21 (23)		
